# Inulin and Freeze-Dried Blueberry Intervention Lead to Changes in the Microbiota and Metabolites within In Vitro Studies and in Cognitive Function within a Small Pilot Trial on Healthy Children

**DOI:** 10.3390/microorganisms12071501

**Published:** 2024-07-22

**Authors:** Buket Horasan Sagbasan, Claire M. Williams, Lynne Bell, Katie L. Barfoot, Carlos Poveda, Gemma E. Walton

**Affiliations:** 1 Department of Food and Nutritional Sciences, University of Reading, Whiteknights, Reading RG6 6AP, UK; bkthorasan@gmail.com (B.H.S.); c.g.povedaturrado@reading.ac.uk (C.P.); 2 Department of Psychology, University of Reading, Earley Gate, Whiteknights, Reading RG6 6AL, UK; claire.williams@reading.ac.uk (C.M.W.); l.bell@reading.ac.uk (L.B.); katie.barfoot@reading.ac.uk (K.L.B.)

**Keywords:** gut microbiota, prebiotic, inulin, flavonoid, anthocyanin, cognition, memory, executive function

## Abstract

The relationship between the gut microbiota and cognitive health is complex and bidirectional, being significantly impacted by our diet. Evidence indicates that polyphenols and inulin can impact cognitive function via various mechanisms, one of which is the gut microbiota. In this study, effects of a wild blueberry treatment (WBB) and enriched chicory inulin powder were investigated both in vitro and in vivo. Gut microbiota composition and metabolites, including neurotransmitters, were assessed upon faecal microbial fermentation of WBB and inulin in a gut model system. Secondly, microbiota changes and cognitive function were assessed in children within a small pilot (*n* = 13) trial comparing WBB, inulin, and a maltodextrin placebo, via a series of tests measuring executive function and memory function, with faecal sampling at baseline, 4 weeks post-intervention and after a 4 week washout period. Both WBB and inulin led to microbial changes and increases in levels of short chain fatty acids in vitro. In vivo significant improvements in executive function and memory were observed following inulin and WBB consumption as compared to placebo. Cognitive benefits were accompanied by significant increases in *Faecalibacterium prausnitzii* in the inulin group, while in the WBB group, Bacteroidetes significantly increased and Firmicutes significantly decreased (*p* < 0.05). As such, WBB and inulin both impact the microbiota and may impact cognitive function via different gut-related or other mechanisms. This study highlights the important influence of diet on cognitive function that could, in part, be mediated by the gut microbiota.

## 1. Introduction

The human gut microbiota varies throughout the gastrointestinal tract and is influenced by several factors, including age, dietary habits, lifestyle, and antibiotic consumption. The gut microbiota is considered to have bidirectional communications with the brain, hence the term gut–brain axis (GBA) [1]. The current understanding is that within this pathway, the gut microbiota release immune activating and other signalling molecules, such as short chain fatty acids (SCFAs), and are involved in the production of neurotransmitters, such as gamma-aminobutyric acid (GABA), serotonin, and biologically active forms of catecholamines in the lumen of the gut [2]. These microbial end products could influence gut/brain interactions and affect several cognitive disorders [3], such as attention deficit hyperactivity disorder (ADHD), autism spectrum disorder (ASD), depressive disorder, and Alzheimer’s disease [4].

Habitual diet can impact the gut microbiota and, as such, there lies the possibility to affect cognitive function via the GBA through the diet. Specific foods targeting the gut microbiota include pre- and probiotics. A prebiotic is a “substrate that is selectively utilised by host microorganisms conferring a health benefit” [5]. As such, a prebiotic is able to positively influence the balance of gut bacteria and/or metabolites and may therefore be able to impact neuro-regulating parameters. Inulin is a known prebiotic, a non-digestible oligosaccharide that can be found in plants, fruits, and vegetables and is selectively used by the gut bacteria, often resulting in increases in bifidobacteria. Furthermore, inulin has been observed to impact mood state in a working population, thus highlighting the potential for inulin to impact the GBA [6]. As such, the impact of inulin on cognitive function is worthy of attention.

When considering other dietary ingredients that may impact the brain, it is worth considering flavonoid-rich foods. Flavonoids are polyphenolic compounds found in many plant-based foods. Evidence indicates that flavonoid containing foods, particularly berry fruits, might be beneficial to several cognitive domains, including attention, working memory, and executive function [7]. Berries, for example, contain a wide range of different flavonoid subclasses but are especially rich in anthocyanins. The impact of berries on cognitive performance has been investigated using freeze-dried blueberries and fresh whole blueberries [8]. Studies of single-dose freeze-dried blueberry interventions have reported positive impacts on attention, inhibition, visuospatial memory, and executive function between 2–6 h post-consumption in 8–10 year old children [9]. Additionally, administration of berry fruits (rich in flavonoids) for 1.5–8 weeks has been linked to improved visuospatial memory and improved long-term memory [10,11]. Several different mechanisms of action have been proposed to explain the positive effects of flavonoids on cognition, including increases in cerebral blood flow, protecting against neuronal stress via anti-inflammatory and anti-oxidative effects, and positively stimulating neural signalling pathways [10,12]. Further to this, flavonoids are known to accumulate in the large intestinal lumen in the mM range and might have an impact on the gut microbial community [13]. Indeed, a dietary intervention human study with wild blueberries demonstrated significant increases in *Bifidobacterium* spp. compared to a placebo drink [14]. It is worth noting that there are many other active ingredients in blueberries that could have an impact on the microbiota and cognitive function, such as fibre. Taken together, these data suggest that regular consumption of a wild blueberry drink could positively modulate the composition of the intestinal microbiota [15,16], thereby impacting cognition and making this a worthy area for investigation.

The prebiotic-like effects of flavonoid interventions might also be based on their antioxidative effects on the gut microbiota [17]. Current evidence suggests that anthocyanins may assist in the maintenance of gut wall integrity, promoting health benefits. The catabolism of anthocyanin cyanidin-3-glucoside (C3G) results in the release of certain phenolic compounds, including protocatechuic acid, vanillic acid, phloroglucinaldehyde, and ferulic acid, all of which have an effect on oxidative stress and inflammation in the gut [18]. In vitro, it has been observed that C3G is hydrolysed to aglycones by enzymes in the small intestine, and further degraded to phenolic compounds by gut microbiota in the colon. As such, C3G and its metabolites play a role in reducing inflammation and oxidative stress in the gut, which helps to provide optimal conditions for metabolism to occur [19]. 

To date, studies on prebiotics and cognitive function are limited. However, in a study by Berding et al. (2021), polydextrose consumption, in healthy adults, led to increases in the executive function (EF) domain of cognition, concurrent with changes to the microbiota [15]. The study associated this outcome with anti-inflammatory effects, supporting the potential role of prebiotics impacting the GBA and aiding cognitive function. Probiotic intake may also positively impact EF [20]. EF defines cognitive processes such as working memory, planning, problem-solving, cognitive flexibility, inhibitory control, and the ability to direct attention and thoughts [21]. The frontal areas of the brain are where the EF is derived, developing and maturing during childhood and into early adulthood [22]. Any disorders in the frontal region during development could have a long-term impact, therefore it is critical to support optimal cognitive development in the early years of life [23]. As such, targeting gut-microbial interventions on a young cohort could support the EF function and development. As a first step, in vitro models of the large intestine can provide a useful tool to determine what impact a dietary ingredient is likely to have on the gut microbial community. In addition to this, the models enable the monitoring of metabolites produced that could be relevant to cognitive health. The combination of these parameters enables more information to be gathered on potential mechanisms of GBA.

The aim of this small study was to use both in vitro models of the large intestine and a pilot study in healthy children to investigate the prebiotic potential, alongside the cognitive and behavioural effects, of a 4-week supplementation of a wild blueberry preparation rich in flavonoids and of inulin. By studying both foods together, it is also possible to consider whether any effects on cognitive function happen through the same mechanisms. An age group of 7–10 years was chosen, as this is a stage of development that involves a significant growth in the frontal lobes of the brain, and the participants may be particularly sensitive to dietary manipulations such as this.

## 2. Materials and Methods

All analytical grade chemicals and gut model media components were purchased from Sigma-Aldrich (Merck), Dorset, UK. Neuroactive standards were obtained from Sigma-Aldrich (Merck), Dorset, UK. Inulin powder, in the form of Orafti^®^Synergy1 was supplied by Beneo-GmbH, Obrigheim, Germany, and the freeze-dried blueberry powder product was supplied by the Wild Blueberry Association of North America, Old Town, ME, USA (Table 1). Oligosaccharide probes for Flow-FISH analysis were purchased from Eurofins, Wolverhampton, UK. For neurotransmitter analysis, HPLC Plus grade acetonitrile (99.9%) was purchased from Sigma-Aldrich (Kent, UK). Formic acid (99% LC/MS grade, HiPerSolv CHROMANORM^®^) was purchased from VWR, Lutterworth, UK. A centrifuge tube filter (Corning^®^ Costar^®^ Spin-X^®^, 0.22 µm Pore CA Membrane, Sterile, 96/Case, Polypropylene) from Sigma-Aldrich (Merck), Dorset, UK, was used to filter batch culture and gut model fluid samples. Analytical standards (LC-MS grade) of dopamine hydrochloride (99%), L (-)-Epinephrine (99%), L-Noradrenaline (98%), and gamma-aminobutyric acid (99%) were from Alfa Aesar (Lancashire, UK).

Polyphenol analyses of freeze-dried blueberry powder were conducted by FutureCeuticals, Momence, IL, USA, and the vitamin, sugar, and dietary fibre analyses were conducted by RSSL, Reading, UK.

### 2.1. Pre-Digestion

An upper gut in vitro digestion was performed on the freeze-dried blueberry (Wild Blueberry; WBB) preparation to yield the non-digestible portion. Test substrate (WBB) was prepared for pre-digestion by combining with distilled water according to the method of Mills et al. (2008) [24]. An amount of 12 g of WBB was taken and 30 mL distilled water added to obtain the pre-digestion solution. The solution was stomached (Stomacher 400 circulator, Seward) for 5 min at 240 paddle beats/min. The oral phase was imitated by combining the stomached substrate solution with α-amylase (4 mg) dissolved in CaCl_2_ (0.001 mol L^−1^, pH 7; 1.25 mL). Substrates were incubated at 37 °C on a shaker (Gyrotory shaker, New Brunswick, St. Albans, UK) for 30 min. For the gastric phase, vessels were reduced to pH 2 using HCl (6 mol L^−1^). The stomached substrate solutions were combined with pepsin (0.54 g) dissolved in HCl (0.1 mol L^−1^; 5 mL). Vessels were incubated at 37 °C on a shaker (Gyratory shaker, New Brunswick) for 2 h. The small intestinal phase was imitated by combining the stomached substrate solution with porcine pancreatin (112 mg) and bile (0.7 g) dissolved in NaHCO_3_ (0.5 mol L^−1^; 25 mL). Vessels were adjusted to pH 7 using NaOH (6 mol L^−1^) and the resulting mixture was incubated at 37 °C on a shaker (Gyratory shaker, New Brunswick) for three hours. The solution was dialysed against NaCl (0.01 mol L^−1^) for 15 h using 500 Da molecular weight cut-off regenerated cellulose tubing (Spectra/Por^®^ 6, Spectrum Europe, Breda, The Netherlands). The solution was then changed, and dialysis proceeded for a further 2 h. The dialysed product was freeze-dried for five days and the resultant powder used for in vitro fermentation. 

### 2.2. Three-Stage Continuous Culture Gut-Model System

Physicochemical conditions in the colon were replicated in a continuous culture system, comprised of three connected fermentation vessels (V) representing the proximal (V1, 80 mL, pH = 5.5), transverse (V2, 100 mL, pH = 6.2), and distal colon (V3, 120 mL, pH = 6.8). A small-scale version of the validated system, described by Macfarlane et al. (1998) [25], was used in this study. 

The growth medium was prepared in distilled water and consisted of per L: 5 g starch, 5 g peptone water, 5 g tryptone, 4.5 g yeast extract, 4.5 g NaCl, 4.5 g KCl, 4 g mucin (porcine gastric type III), 3 g casein, 2 g pectin (citrus), 2 g xylan (oatspelt), 2 g arabinogalactan (larch wood), 1.5 g NaHCO_3_, 1.25 g MgSO_4_.7H_2_O, 1 g guar gum, 1 g inulin, 0.8 g cysteine, 0.5 g KH_2_PO_4_, 0.5 g K_2_HPO_4_, 0.4 g bile salts No. 3, 0.15 g CaCl_2_.6H_2_O, 0.005 g FeSO_4_.7H_2_O, 0.05 g hemin, 10 mL Vitamin K, and 1 mL Tween 80. 

Faecal samples were obtained from three healthy donors aged 7–10 years. The donors were free of any metabolic and gastrointestinal diseases, were not taking probiotic or prebiotic supplements and had not taken antibiotics 6 months before faecal sample donation. None of the children followed any specific or restricted diet. All parents provided written informed consent for use of their children’s faeces in this study. This study was approved by The University of Reading research Ethics Committee (UREC 15/20). Faecal samples were placed in an anaerobic jar (AnaeroJarTM 2.5 L, Oxoid Ltd., Basingstoke, UK) including a gas-generating kit (AnaeroGenTM, Oxoid, Basingstoke, UK). An aliquot of 20 g of an individual sample was diluted in 100 mL anaerobic PBS (0.1 mol/L phosphate buffer solution, pH 7.4, *w*/*w*) and homogenised (Stomacher 400, Seward, West Sussex, UK) for 2 min at 240 paddle beats per min. The sample was then added to two anaerobic fermenters within 2 h of production (WBB and inulin model). This process was carried out three times, using a different sample each time. The pH of each vessel was maintained using a pH pump (Electrolab, Tewkesbury, UK) with 1 M NaOH and 1 M HCl solutions, as appropriate. An anaerobic environment was maintained by continuous sparging with oxygen-free nitrogen (15 mL/min). Following inoculation, the system was run for 24 h to allow the bacteria to multiply within the vessels. Then, the continuous flow was started with a retention time of 36 h, which is appropriate for children at the age of 7–10 [26]. The system was run for at least 8 full volume turnovers to allow for the gut bacteria and the metabolites to reach equilibrium. When the SCFA profile stabilised (+/− 10%), steady state 1 was achieved (SS1) before starting inulin or pre-digested freeze-dried blueberry administration. Taking into account the operating volume (300 mL) and the retention time (36 h, flow rate 8–9 mL/h) of the colonic model system, after reaching SS1 (12 days), inulin (synergy 1) was added daily into V1 at a dose of 0.6 g, equivalent to 5–8 g of inulin/d in the diet, which is considered an appropriate prebiotic dose [27], or pre-digested freeze-dried blueberries (WBB) (1 g/daily) (in total, 12 g of WBB). Considering pre-digestion and the whole gut model system, this is equivalent to approximately 240 g fresh wild blueberries, containing 253 mg of anthocyanins [28]. The substrates were added to the system on a daily basis for at least a further 8 volume turnovers, upon which a second equilibrium was achieved (steady state 2 (SS2)) (12 days). Aliquots of 5 mL were removed at SS1 and SS2 to assess changes in microbes and metabolites. Each colonic model system took 24 days to complete.

### 2.3. Bacterial Enumerations by FISH-FCM (Fluorescence In Situ Hybridisation with Flow Cytometry)

Fluorescence in situ hybridisation (FISH), coupled with flow cytometry, was used to determine changes in the microbial community within the gut models [29]. The oligonucleotide probes were commercially synthesised and labelled at the 5′ end with the fluorescent dye Cy3 (Eurofins Genomics, UK), as reported in Table 2. The probes targeted functionally important groups of bacteria to determine how the treatments impacted these.

### 2.4. SCFA Analysis

Levels of SCFAs were derivatised for analysis by GC, as previously described by Richardson et al. (1989) [40]. A GC Agilent 7890B gas chromatograph (Agilent, Cheshire, UK) using an HP-5 MS (L × I.D. 30 m × 0.25 mm, 0.25 µm film thickness) coating of crosslinked (5%-phenyl)-methylpolysiloxane (Hewlett Packard, Reading, UK) was used for SCFA detection. An amount of 1 µL of each sample was injected, with a run time of 17.7 min. The injector and detector temperatures were 275 °C, and the column temperature was programmed from 63 °C to 190 °C and held at 190 °C for 30 min. Helium was the carrier gas (flow rate, 1.7 mL/min, head pressure, 133 KPa). Peaks were integrated using Agilent Chem Station software 2.3.53 (Agilent Technologies, Basingstoke, UK), and the SCFA content was quantified by the single-point internal standard method [41]. Peak identity and internal response factors were determined using a 20 mM calibration cocktail, including acetic, propionic, and butyric acids.

### 2.5. Neurotransmitter Analysis

The concentration of neuroactive metabolites was determined by Liquid Chromatography Mass Spectrometry (LCMS), following the method of Zhai et al. (2015) [42], with further modifications. Samples were taken from the gut model system at SS1 and SS2 from proximal (V1), transverse (V2), and distal (V3) vessels and were centrifuged at 11,200× *g* (SANYO MSE Mistral 3000i, Sanyo Gallenkamp PLC, Cambridge, UK) for 3 min. An amount of 400 µL supernatant was taken and centrifuged again at 11,200× *g* for 3 min with filtered centrifuge tubes (Corning Costar centrifuge tube, filter acetate membrane-0.22 µm). A total of 200 µL of HPLC water (Blank), calibration-standard samples and gut-model samples were placed in 96-well plates.

Separate standard stock solutions (10,000 ng/mL) of 5 analytes, including serotonin (5-HT), dopamine (DA), gamma-aminobutyric acid (GABA), norepinephrine (NE) and epinephrine (EPI), were individually prepared in HPLC water. A 1000 ng/mL mixed standard solution containing the 5 analytes was made by acquiring aliquots of each separate stock solution. The mixed standard solution was appropriately diluted with HPLC water to prepare a calibration series. A calibration series of spiked standard samples was prepared including 9 levels: 1, 10, 50, 100, 250, 500, 750, 1000, and 2500 ng/mL.

Chromatographic separation was performed on an Agilent C18 column (250 × 4.6 mm, 5 μm; Agilent Co., Ltd., Santa Clara, CA, USA) with a protected C18 column cartridge (4.6 × 12.5 mm cartridge, 5 μm; Agilent Company, USA). The column temperature was kept at 25 °C. The injection volume was 10 μL. The mobile phase consisted of A (0.1% formic acid + HPLC water) and B (0.1% formic acid + acetonitrile (ACN)), with gradient elution as follows: 0–2 min, 100%; 2–5 min, 75% A; 11–15 min, 65% A; 15–20 min, 5% A; and then within 0.1 min, it returned to initial 100% A. This was followed by the equilibration period of 5 min prior to the injection of each sample. The flow rate was set to 0.25 mL/min.

The LC/MS-8050 triple quadrupole (QQQ) detector was operated in multiple reaction monitoring (MRM) mode using the polarity-switching electrospray ionisation (ESI) mode. Optimal conditions were as follows: dry gas temperature 300 °C, dry gas flow rate of 10.0 L/min. An amount of 4 µL of samples were injected. Samples were measured as the target compounds based on MRM. For the analysis of primary metabolites 5-HT, DA, GABA, NE, and EPI, the LC/MS Method Package for Primary Metabolites (Shimadzu Corporation, Kyoto, Japan) was used.

A linear calibration curve was generated based on the detected signal, proportional to the concentration of the analyte. Good linearity, with an R^2^ greater than 0.98, was obtained across the set calibration in the range from 1 ng/mL to 1000 ng/mL for each of the analytes, with accuracy within 100% ± 20%. Quantification of samples was determined by calibration with 5 analytes, including 5-HT, DA, EPI, GABA, and NE. Samples were measured using online Nexera LC System coupled to LCMS-8050 triple quadrupole (QQQ) mass spectrometry (Shimadzu Corporation, Kyoto, Japan). Data were processed using LabSolutions LCMS version 5.65 software.

### 2.6. Human Intervention Study

The intervention study was reviewed and given a favourable ethical opinion to proceed by the University of Reading Research Ethics Committee (UREC 15/10, UREC 15/58) and was conducted in accordance with the Declaration of Helsinki and Human Tissue Act 2004. The freeze-dried blueberry powder product was supplied by the Wild Blueberry Association of North America. The anthocyanin content of the blueberry powder was analysed as detailed in Table 1. Inulin (Orafti^®^ Synergy 1) and maltodextrin powder products were supplied by BENEO GmbH, Germany.

For the pilot study, 13 participants aged 7–10 years (M = 8.54 years, SD 1.27) were recruited from local primary schools in the Berkshire area, UK, and were randomised to a treatment. Two participants withdrew from this study (reason: preferred not to continue), making the total participant number 13 (6 female, 7 male). Written consent was obtained from parents or legal guardians in advance of the child’s participation. On initial recruitment, parents or legal guardians confirmed that the children spoke English as a first language, had not been diagnosed with ADHD or any other cognitive disorders, and had no known fruit or fruit juice intolerance. Participants were asked to maintain their normal diet during this study. Demographic details of the participants are shown in Table 3. No participant had any known health conditions, e.g., diabetes, obesity, high blood pressure, thyroid, kidney or liver diseases, or psychological diagnoses, e.g., ASD. Participants had not taken any antibiotics for 6 months before this study. Participants were advised not to take any flavonoid supplements, prebiotics, or probiotics during this study.

Three subsets of the British Ability Scale 3, including matrices, pattern construction, and verbal similarities, were used to calculate an overall measure of general ability of the children at baseline [27].

Participants were assigned to receive either WBB (*n* = 4), inulin (*n* = 5), or maltodextrin (*n* = 4), according to a randomised, double-blind, parallel-group design, over 4 weeks. The randomisation was performed through an online tool, sealedenvelope.com. All interventions were weighed, packaged (sachets), and stored at −18 °C by a researcher in the University of Reading School of Psychology and Clinical Language Science. Children received a daily dose of either a 13.3 g WBB (253 mg anthocyanins; equivalent to 240 g fresh blueberries), or a prebiotic supplement (5 g of an oligofructose-enriched chicory inulin product, BENEO Orafti^®^Synergy1), or a placebo-control (5 g of maltodextrin, BENEO, Obrigheim, Germany), administered in 170 mL of water, with 30 mL of a flavonoid-free orange squash concentrate (Rocks Orange Squash, Rocks Drinks Limited, Exeter, UK). Participants’ parents/guardians were issued with 28 daily sachets, alongside a 500 mL bottle of Rocks Orange Squash, a 30 mL measurer, and an opaque shaker flask with instructions on how to prepare the intervention. The drinks were prepared fresh each day by the participants’ parents. The participants only saw the drink once it was in the opaque shaker cups, when it was ready to consume, in order to maintain blinding procedures.

**Figure 1:**
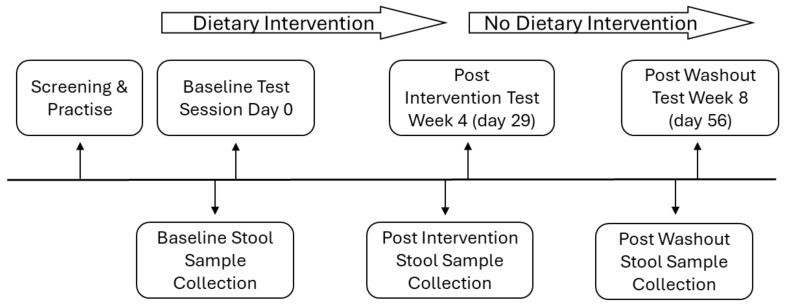
Study design comprising cognitive and stool data collection at baseline (week 0), post-intervention (week 4), and post washout (week 8).

Participants performed cognitive tests and provided a stool sample at the beginning of this study (baseline period), and after 4 weeks (post-intervention). After the intervention period (participants were requested not to use any supplements for 4 weeks), cognitive tests were performed again, and stool samples were provided after those additional 4 weeks (post-washout). Measures of cognition were the primary outcomes, and measures of gut microbiota composition were secondary (see Figure 1). Testing took place outside of school hours (3–5 p.m.) to minimise organisational demands and to maximise the cognitive demand on participants. Interventions were prepared daily at the participants’ house by the participants’ parent(s) or guardian(s), to be consumed early in the morning with breakfast. To measure compliance parent(s) or guardian(s) completed drink consumption logs stating the daily time of study drink consumption.

### 2.7. Cognitive Tests

The cognitive tests were presented via E-Prime V3 (Psychology Software Tools, Inc., Sharpsburg, PA, USA), with participants wearing enclosed headphones throughout all tasks.

Rey’s auditory verbal learning task (RAVLT) was used to assess learning, memory recall, and word recognition. Participants were presented with an auditory recording of 15 nouns (list A). Each presentation was followed by a free recall of this list (Recalls A1–A5). A further list of fifteen nouns (list B) was then presented as an interference list and recalled once only (Recall B). There was a further free recall of list A (Recall A6), followed by a 15-min delay and then a final free recall of list A (Recall A7). After recall A7, participants were visually presented with 50 nouns containing the words from lists A and B, and 20 additional distractor nouns. They were asked to press the ‘yes’ button (keyboard B) for any words from list A and the ‘no’ button (keyboard N) for any words not in List A (including List B or distractor words). The number of correct answers were represented as “Word recognition”. For each test session, the following outcomes were calculated, as specified in Lezak et al. (2012) [44]: immediate word span (Recall A1), showing immediate free recall ability; number of words learned (Recall 5) minus Recall 1 (A5-A1), showing learning over the session; final acquisition (Recall A5), showing the total number of words learned; delayed word recall (Recall A7), showing the ability to recall specific information acquired earlier [45]. Outcomes, including word span (A1), final acquisition (A5), total acquisition (A1-A5), amount learned (A5-A1), and delayed recall (A7) were recorded.

The modified attention network task is a modification of the flanker task (MANT). The MANT measures executive function, attention, and inhibition, providing outcome measures of accuracy (proportion of correct responses, 0–1) and reaction time (RT) for correct responses. The direction of a central arrow is responded to by participants (using keyboard arrows). Presentation conditions can either be congruent, incongruent, or neutral, depending on the direction of any arrows surrounding the central arrow. Load is manipulated by varying the number of surrounding arrows. Accuracy and response times for each treatment were measured for each congruency and load condition. The combination of speed and accuracy measures provides an indication of the participant’s attention and inhibitory control. Full details of both the RAVLT and the MANT can be seen in Barfoot et al. (2019) [46].

### 2.8. Faecal Microbial Analysis

Stool samples were collected from participants, and the faecal microbiota were assessed by 16S ribosomal RNA (rRNA) amplicon sequencing to characterise the bacterial groups present and their abundance.

A 250 mg quantity of faecal pellet was weighed according to the DNeasy PowerSoil Kit (Qiagen, Manchester, UK) extraction kit protocol. The samples were homogenised by vortex in the PowerBead tube provided. The subsequent steps of DNA extraction were performed per protocol. The DNA yield was measured using a NanoDropH ND-1000 UV spectrophotometer (Nano-Drop Technologies, Wilmington, DE, USA). Primers were used to amplify the V3/V4 variable region of the 16S rRNA gene for the profiling of the bacterial faecal microbiota performed by Eurofins Genomics Europe Sequencing GmbH. For each sequence read, low quality calls were removed before further processing using a sliding window approach. Thresholds used for sequence quality filtering had a value length threshold of 221 and a quality threshold of 10. To assemble and cluster, paired-end amplicons were sequenced in both directions. The resulting read pairs were merged based on overlapping bases using FLASh, with a maximum mismatch density of 0.25. Sequence data were compressed by clustering using cd-hit based on 99% similarity, accounting for PCR and sequencing errors (<1%). At a high sequencing depth, each original template was sequenced multiple times. Therefore, clusters containing only one sequence were removed from further analysis. Clustered data were checked for chimeras using UCHIME, and corresponding clusters removed from further analysis. Non-chimeric, unique clusters were subjected to BLASTn analysis using non-redundant 16S rRNA reference sequences with an E-value cutoff of 1 × 10^−6^. Reference 16S rRNA sequences were obtained from the Ribosomal Database Project. All hits to the reference 16S rRNA database were considered, and specific filters applied to the hits to remove false positives (with filter thresholds for identity being over 97% and alignment coverage being over 95%). Only good quality and unique 16SrRNA sequences with a taxonomic assignment were considered and used as a reference database to assign operational taxonomic unit (OTU) status to the clusters. Taxonomic classification was based on the NCBI taxonomy. Classification of OTU clusters and size of OTUs (number of reads within one cluster) were consolidated to compute relative abundancies. The percentage abundance was determined for each OTU by considering all the OTUs in the sample and was used as a criterion (>0.5%) to select highly abundant OTUs in the sample.

### 2.9. Statistical Analysis

#### 2.9.1. In Vitro Continuous Culture

Data from LC-MS, GC, and FCM-FISH were analysed using SPSS (SPSS Statistics version 22). Student paired *t*-tests were used to compare the two time points, SS1 and SS2, to determine the impact of inulin compared to no inulin, or WBB compared to no WBB, this comparison was made in the 3 vessels. Furthermore, the difference between the three vessels and that between the WWB model and the inulin model were ascertained by the Student’s *t*-test. Results were considered significant at *p* ≤ 0.05. Bonferroni adjustment was used for the correction of multiple comparisons.

#### 2.9.2. Human Cognitive Data

The RAVLT and MANT data were analysed by Linear Marginal Models (LMM) using an unstructured covariance matrix to model repeat measures, performed using SPSS (Version 22.0). Separate models were performed for each dependent variable. For RAVLT, outcomes included word span, final acquisition, total acquisition, amount learned, and delayed recall. The MANT outcomes included accuracy and RTs. Baseline performance scores were included as a covariate in the analysis of all cognitive tests. Drink (placebo, inulin, WBB), time (post-intervention, post-washout), and drink × time were included as fixed factors to compare the effects of the treatment across the intervention period. In addition, for the MANT task, congruency (congruent, incongruent, neutral), load (high, medium, and low load), and their respective interactions with drink and time were also included as fixed factors in the model to detect changes in relation to cognitive demand, as in Whyte et al. (2017) [20] and Khalid et al. (2017) [11]. All post-hoc pairwise comparisons were corrected for type 1 errors using Bonferroni adjustment.

#### 2.9.3. Human Microbiota Data

The 16S rRNA gene sequencing data were analysed by LMM, using an unstructured covariance matrix to model repeat measures, performed with SPSS (Version 22.0). Separate models were performed for each dependent variable. The % abundance of each genus and phylum was included as a dependant variable. Baseline performance was included as a covariate. Drink (placebo, inulin, WBB), time (post-intervention, post-washout), and drink × time were included as fixed factors to compare the effects of treatment across the intervention period. All post-hoc pairwise comparisons were corrected for type 1 errors using Bonferroni adjustment.

## 3. Results

### 3.1. In Vitro

#### 3.1.1. Bacterial Enumeration

The changes in bacterial composition following the gut model fermentation are reported in Table 4 and Table 5. Significant increases in *Bifidobacterium* spp. following the administration of inulin were observed in V1 (proximal region) and in V3 (distal). At SS2, the growth of *Bifidobacterium* spp. was significantly higher upon the fermentation of inulin proximally and distally compared to WBB (*p* < 0.01). Moreover, at SS2, the growth of *Bifidobacterium* spp. was significantly higher proximally at SS2 compared to SS1 in the WBB model (*p* < 0.01). Significant increases in the growth of *Lactobacillus* spp. were observed following fermentation of both inulin (*p* < 0.01) and WBB proximally (*p* < 0.05) after treatment (SS2). The growth of *Lactobacillus* spp. increased in the other regions, but this was not significant. Interestingly, at SS2, the growth of *Lactobacillus* spp. was significantly higher in V1 in the WBB model compared to the inulin model in V1 (*p* < 0.05). There was an increase in *Roseburia* spp. following fermentation of inulin distally (*p* < 0.01) and in the WBB model proximally (*p* < 0.05). Additionally, after treatment in the WBB model, the growth of *Roseburia* spp. was significantly lower in the distal region (V3, pH 6.8) compared to the proximal region (V1, pH 5.5), whereas it was also found to be significantly lower compared to the distal region (V3, pH 6.8) in the inulin model (*p* < 0.05). Moreover, there were significant decreases in the growth of Desulfovibrionales and the *Clostridium histolyticum* group following the fermentation of inulin in the proximal regions (V1; *p* < 0.05).

#### 3.1.2. Short-Chain Fatty Acids (SCFA)

**Figure 2:**
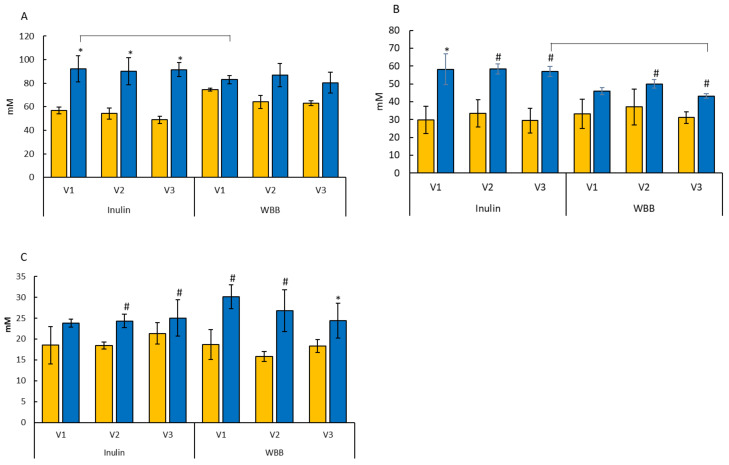
SCFA concentrations. (**A**) Acetate, (**B**) butyrate, (**C**) propionate concentrations within in vitro gut model systems before (SS1 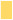) and after (SS2 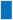) administration of inulin (INU, 0.6 g/d) and freeze-dried blueberries (WBB, 1 g/d). (*n* = 3): healthy child faecal donors. Values are mean ± SD (mM). Significant difference in each vessel: * *p* < 0.05; # denotes a trend *p* < 0.1; brackets indicate a significant difference compared to other treatment.

The SCFA concentrations are shown in Figure 2. Significant increases were observed in acetate after the administration (SS1 vs. SS2) of inulin in all vessels (V1, V2, and V3) (*p* < 0.05), whereas no significant differences were observed after the administration of WBB. Supplementation of inulin led to a significant increase in the butyrate concentration in the vessels modelling the proximal (V1) region (*p* < 0.05), with trends of increase in the transverse (V2) and distal (V3) regions (*p* < 0.1). Similarly, WBB also led to a trend of increase in the butyrate concentration in the vessel modelling the transverse (V2) and distal (V3) colonic regions (*p* < 0.1). WBB led to a significant increase in the propionate concentration in the distal (V3) part of the colon after treatment (SS2), whilst trends of increase were observed in the proximal (V1) and the transverse (V2) regions (*p* < 0.1). Trends of increase were also observed in propionate concentration following the fermentation of inulin in the transverse (V2) and the distal (V3) regions (*p* < 0.1). Acetate and butyrate were the main end products after the administration of both inulin and blueberries.

#### 3.1.3. Neurotransmitters

**Figure 3:**
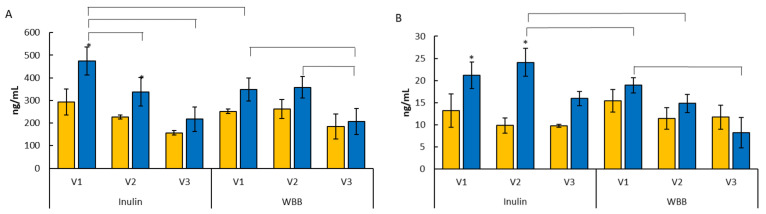
GABA (**A**) and serotonin (**B**) concentrations determined by LC-MS; * denotes significantly different compared to SS1; a bracket indicates significantly different compared to the other vessel or treatment (SS1 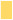) and after (SS2 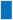).

The neurotransmitter concentrations are shown in Figure 3. Significant increases were observed in GABA production following the fermentation of inulin between the SS1 and SS2 time points in the proximal (V1) and the transverse regions (V2) (*p* < 0.05). Furthermore, the production of GABA was significantly higher in the inulin model compared to the WBB model in the proximal region (V1, pH 5.5) (*p* < 0.05). The amount of GABA was significantly higher in the proximal region (V1, pH 5.5) and in the transverse region (V2, pH 6.2) compared to the distal region (V3, pH 6.8) in the inulin model. The levels of GABA were also observed to be significantly higher in the proximal region compared to the distal region (V3, pH 6.8) upon the fermentation of WBB (*p* < 0.05). A significant increase in serotonin production was observed following inulin fermentation in the proximal regions (V1) (*p* < 0.01) and transverse regions (V2) (*p* < 0.01). Additionally, the serotonin concentration was significantly higher in the V2 compared to the V3 region in the inulin model, whilst in the WBB model, serotonin was higher in V1 compared to V3 (*p* < 0.05). Moreover, the production of serotonin was found significantly higher in the inulin model in the V1 and V2 regions compared to the WBB model in the V2 region (*p* < 0.05). The levels of dopamine, norepinephrine, and epinephrine were non-detectable.

### 3.2. In Vivo Pilot Study

#### 3.2.1. Cognitive Changes

The changes in cognitive behaviour are shown in Table 6. Both the inulin and the WBB groups displayed significantly improved delayed recall memory performance compared to the placebo treatment. These active treatment groups also learnt more words compared to those participants administered the placebo, showing better final acquisition after the 4-week intervention period, an effect which was maintained even after the 4-week washout period (*p* < 0.05). The inulin group also performed better at total acquisition performance compared to the placebo group. No significant WBB and/or inulin-related effects were observed on the word recognition measures of this task at any timepoint. Post-intervention and washout WBB and inulin-related benefits were also seen on the modified attention network task (MANT), a task measuring EF. The inulin group maintained a significantly higher accuracy on congruent medium-load trials compared to the placebo-treated participants (*p* < 0.05) after the 4-week intervention, whilst following the washout period (4 weeks after the intervention), significant increases in accuracy on congruent high-load trials for the WBB group compared to the placebo group were maintained (*p* < 0.05). No significant differences in RT were observed for either the WBB or inulin groups compared to placebo.

#### 3.2.2. Microbiota Changes

**Figure 4:**
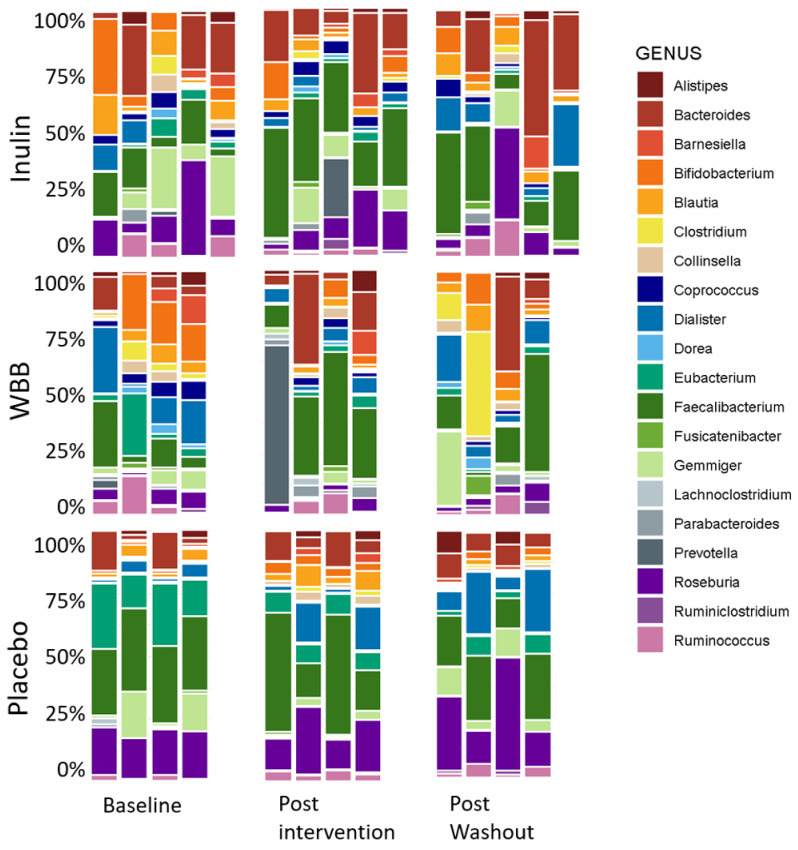
Microbiome (genus) composition (% abundance) at genus level in participants during intervention with inulin, WBB, and placebo (maltodextrin) at baseline, post-intervention, and post washout periods. Results are reported as % abundance of the data (each stacked bar represents a different participant).

**Figure 5:**
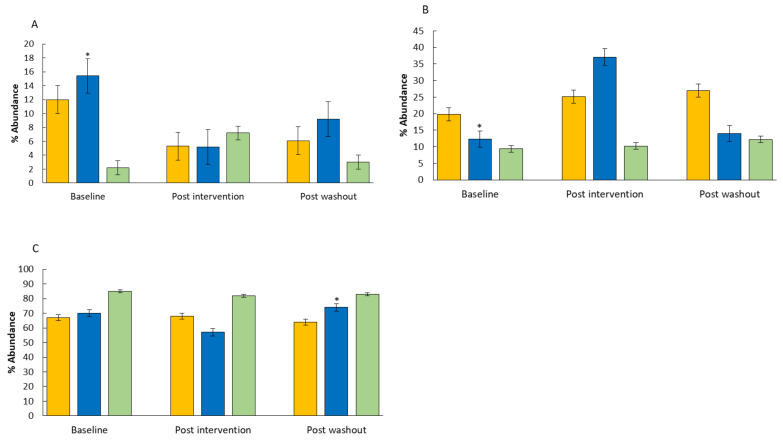
Microbiome (phylum) composition (% abundance). (**A**)—Actinobacteria, (**B**)—Bacteroidetes, (**C**)—Firmicutes; during intervention with inulin 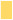, WBB 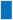, and placebo 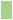 (maltodextrin) at baseline, post-consumption, and post-washout. Results are reported as means (% abundance) of the data ± SD (*n* = 5 (inulin), *n* = 4 (WBB), *n* = 4 (placebo)). Significant differences in each group * *p* < 0.05; significantly different compared to post-intervention.

The changes in the abundance of the bacterial genus and phylum levels are displayed in Figure 4 and Figure 5. No significant changes were observed in *Alistipes* spp. and in *Bifidobacterium* spp. levels. However, at baseline the abundance of *Bifidobacterium* was significantly higher in the WBB group compared to the placebo group. A significant increase in the abundance of *Faecalibacterium prausnitzii* was seen in the inulin group post-treatment (*p* < 0.05) compared to the placebo, whilst an increasing trend was observed in the WBB group post-intervention (*p* < 0.1). Additionally, the abundance of *F. prausnitzii* was significantly lower in the inulin and the WBB groups compared to the placebo group at the beginning of this study. There was a significant decrease in *Blautia* spp. following consumption of inulin compared to the placebo (*p* < 0.05). Interestingly, the abundance of *Clostridium* spp. in the WBB group increased significantly after the intervention, and the abundance of *Clostridium* spp. was significantly higher in the WBB group after the washout period (*p* < 0.05), whilst no significant changes were observed in the placebo and inulin groups. The abundance of the *Coprococcus* spp. decreased significantly post-intervention and post-washout in the WBB group compared to the placebo group (*p* < 0.05). In addition, the abundance of *Coprococcus* spp. was significantly higher pre-intervention both in the inulin and the WBB group compared to the placebo group. Alterations in the abundance of the bacterial phylum are shown in Figure 5. There were significant changes in the WBB group in the abundance of phylum post-consumption. As such, a significant decrease was observed in Actinobacteria (*p* < 0.05), and a significant increase was observed in Bacteroidetes (*p* < 0.05), whilst no significant changes were seen either in the placebo or in the inulin groups. Additionally, there were significant differences in the abundances of *Roseburia* spp. and *Bifidobacterium* spp. between the placebo and the WBB groups at the beginning of this study (*p* < 0.05).

## 4. Discussion

The current study was conducted to explore the impact of inulin and blueberries on cognitive function parameters in children and on the faecal microbiota and metabolite production. In vivo and in vitro approaches were combined to provide both functional and mechanistic data. In the pilot study, a daily dose of flavonoid-rich wild blueberry (WBB) (13.3 g), prebiotic inulin (5 g), or a placebo (maltodextrin) (5 g) was administered to a small group of typically developing 7–10-year-old children over 4 weeks to investigate the cognitive benefits and the impact on the gut microbiota linked with the gut–brain axis. In vitro, the equivalent dose of WBB and inulin was provided to gut model systems inoculated with faeces from healthy children (7–10 years). Samples were taken to determine changes in the microbial community and in metabolites, including neurotransmitters.

In vivo, both the inulin and the WBB intervention led to a significantly improved performance on the delayed word recall and final word acquisition measures compared to the placebo. The current results match well with the findings of Whyte and Williams (2015), as they reported the ability of WBB to improve episodic memory performance, with significant improvements in delayed recall memory [9]. These findings add to the literature indicating that auditory recall memory measures may be sensitive to anthocyanin interventions. In a previous study in older adults (65–80 years), WBB supplementation enhanced immediate recall in an auditory verbal learning task, concurrent with improvements in vascular function; however, no microbial changes were observed. The mechanisms of action of WBB were therefore considered to be associated with an increase in cerebral blood flow (CBF) or by facilitating the up-regulation of brain-derived neurotrophic factor (BDNF) [47]. Similarly, improved recall performance in an inulin study was also attributed to non-microbiota mechanisms. Smith et al. (2015) observed greater accuracy on a recognition memory task, and improved recall performance following acute consumption of inulin [27]. The effects were observed two hours after the ingestion of the inulin, and therefore were acute. Therefore, in this case, the impact of inulin acting as a prebiotic (i.e., via the gut microbiota) was ruled out. As such, different mechanisms could underlie these acute effects. Findings in the literature were generally based on acute effects, which can rule out the prebiotic effects driving the changes observed in those studies. However, in the current study, by having a 4-week intervention, there is also the potential for effects to have been derived from changes in the microbiota.

Potential improvements were seen in both active treatment groups in the EF domains of cognition. Indeed, significantly higher accuracy was seen in the MANT in congruent medium-load trials in the inulin group compared to the placebo group, whereas significantly higher accuracy was also seen in the congruent high-load trials in the WBB group following washout, compared to the placebo group. In the inulin group, these changes were concurrent with increases in the abundance of the faecal bacteria *Faecalibacterium prausnitzii*. Whilst this microbial change was not observed within the in vitro models, previous interventions with inulin in adults (*n* = 12) have reported significant increases in *F. prausnitzii* during the period of inulin consumption (10 g/d over 16 days) [48]. *F. prausnitzii* have been reported to improve cognitive impairment in a mouse model for Alzheimer’s disease (AD) [49]. *F. prausnitzii* is a dominant bacterial species detected in the healthy human large intestine and a known butyrate producer [50]. In the in vitro study, inulin fermentation led to enhanced levels of butyrate. Butyrate has been considered to have a positive impact on cognitive and memory functions [51]. As such, this pattern of change is likely to positively impact cognitive function.

In the intervention study, consumption of WBB resulted in a significant reduction in the Firmicutes/Bacteroidetes (F/B) ratio, owing to a significant increase in the abundance of Bacteroidetes post-consumption. Several studies have reported that phenolic acids and flavonoids reduce the F/B ratio, which could be also associated with protection against cognition disorders and obesity [52,53]. A study known as the Canadian Healthy Infant Longitudinal Development (CHILD), performed with 405 infants (199 females), determined a link between the gut microbiota and neurodevelopment in a general population birth cohort [54]. The child participants, aged 1–2 years old, were assessed for their neurodevelopmental outcomes using the Bayley Scale of Infant Development (BSID-III). The Bacteroidetes-dominant cluster was associated with higher scores for cognitive, language, and motor development, at 2 years of age, in models adjusted for covariates. Similarly, in the current study, Bacteroidetes increased following WBB consumption, concomitant with the positive outcomes of executive and memory function. An increasing number of human studies show a relationship between prebiotics and cognitive effects. As such, galactooligosaccharides (GOSs) have been reported to have a significant impact on social behaviour [55], and higher levels of 2′fucosyllactose in infants have been associated with improved EF function at three years of age [56]. However, in terms of congruency in the EF domains of cognition, the current findings differed from other findings in the literature. For example, in the study by Whyte et al. (2017) [21], EF performance in 7–10 year-old children was significantly improved with WBB compared to the placebo, particularly on more cognitively demanding, incongruent and high-load trials in the MANT. Furthermore, Whyte et al. (2016) performed another study with 7–10-year-old children and reported a significant increase in accuracy again in the more demanding incongruent trials whilst consuming WBB [45]. Interestingly, in the current study, significant improvements were only observed in the congruent trials, but not in the incongruent trials following inulin and WBB.

When considering mechanistically whether the microbiota could be involved in some of the changes observed, the in vitro work can provide possible routes. For example, within the in vitro study, significant increases in the levels of the neurotransmitters GABA and serotonin were observed upon the fermentation of inulin. The use of the gut model system allows for the production of neurotransmitters, in the absence of human cells, to be explored. GABA, for instance, is produced by decarboxylation of L-glutamate by glutamic acid decarboxylase (GAD) by the microbiota in response to an acidic environment. Therefore, the observation of increased levels of GABA in the more proximal and acidic vessels of the model system was expected. In the current in vitro study, these changes were concurrent with increases in lactobacilli and bifidobacteria. Both these genera have been observed to produce GABA. For example, in a study screening for genes associated with GABA production, *Bifidobacterium adolescentis* was identified as a high GABA producer [57]. Similarly, lactobacilli have been identified as GABA producers [58]. So, it is likely that the increases in these genera observed in vitro were linked to increased levels of GABA. Whilst it is not fully understood how gut-produced GABA impacts the brain, a study of Leonte et al. (2017) reported that oral GABA administration in adults leads to increased temporal attention [59], so there is a possibility that gut microbially produced GABA could elicit positive effects in this way. Direct serotonin production by bacteria has been observed less frequently, although it is likely that microbial production of serotonin may occur via the decarboxylation of tryptophan to tryptomine. Little is currently known on which bacteria are the key producers; however, in vitro with mixed faecal fermentation, small concentrations have been observed. From the levels observed in the current study and in previous research, the amount produced is unlikely to be sufficient to impact the GBA [60,61]. In the human body, enterochromaffin cells are involved in the pathway of serotonin production, yielding higher concentrations. Serotonin is known to have an impact on an array of biological processes, including vascular function and platelet aggregation [62]; furthermore, the pathway for serotonin production involves dietary tryptophan and the microbiota. Reduced tryptophan levels have been associated with impaired episodic memory in healthy adults [63]. As such, the microbiota role in serotonin production could help drive changes in cognitive function. The same effects on neuro-modulating molecules in vitro were not observed for WBB, so it is likely that the cognitive effects upon intervention with these two substrates are via different modes of action. However, both substrates did lead to alterations in the microbiota and enhanced levels of SCFA, so the GBA impact from inulin and WBB supplementation cannot be ruled out.

In the intervention study, a variation in the baseline values of bacteria was observed. As such, changes observed should be interpreted with caution, particularly when considering the low number of volunteers. However, from the in vitro data, the potential of the substrates to modulate the microbiota can be seen. For example, *Roseburia* spp. levels were significantly higher in the proximal region of the colon as compared to the distal region in the WBB model. *Roseburia* is also a known butyrate-producing genera. The proximal region of the colon has greater nutrient availability; therefore, the proximal colon might have been more optimal for the growth of these genera. This result would not be seen in the faeces, as faeces are more representative of the distal colon. Furthermore, in vitro fermentation of blueberries and inulin were observed to lead to increases in the levels of *Lactobacillus* and *Bifidobacterium*, and this matches well with findings of other researchers [14,16,64,65]. Furthermore, flavan-3-ols have also been reported to increase the growth of *Lactobacillus* spp. in similar fermentation experiments [66]. Changes in these microbial groups are of interest, as they are involved in the production of the neurotransmitter GABA, being also associated with an improved gut-barrier function: two key pathways of the GBA [1]. Changes in these groups were not observed in the intervention study, possibly due to the low volunteer number. Nevertheless, *Bifidobacterium* spp. have been observed to be involved in cross-feeding pathways, whereby the acetate they produce can be used by other organisms, including *F. prausnitzii*, to produce butyrate [67]. Thus, indirect pathways of cross-feeding could also be at play.

A prebiotic, through selective fermentation, results in a positive impact on the microbial community. In this current in vitro work, upon the fermentation of inulin, there was a significant decrease in the *C. histolyticum* group. These are often proteolytic bacteria that can be associated with negative effects, including tumour promoting properties, inflammatory bowel disease, and even autistic spectrum disorder [68,69]. The effects of inulin on this microbial group have been observed before [70]. Furthermore, several batch-culture fermentation studies have reported a significant decrease in the *C. histolyticum* group following the fermentation of grape fibre and wine extracts [71]. Overall, by increasing potentially positive bacteria (*Bifidobacterium* and *Lactobacillus*), whilst decreasing potentially negative organisms (*C. histolyticum* group), inulin fermentation led to a potentially positive microbiota shift, as would be expected from a prebiotic. As a promising finding, in the in vitro study, the berries had a similar effect with regards to *Lactobacillus* and *Bifidobacterium*, combined with an increase in a butyrate-producing genera. Thereby, the impact of WBB on the microbiota seemed to be positive.

It is worth considering the limitations of this research. The results could be affected by several factors. The in vivo study included a very low number of participants. Furthermore, the period of this study, e.g., the 4-week timescale, as compared to other acute studies, would impact the results. The treatment doses and the sensitivity levels of each cognitive tests could all affect the outcomes. Differences might also be a consequence of the lifestyles of each individual, particularly considering diets resulting in differences in microbiota between the groups. As such, homogeneity among groups would be important for detecting differences. Nevertheless, the data do give us an insight into how nutrition interventions may impact cognitive function, and which factors are important to consider. In vitro, the lack of human cells and absorption means that the neuro-active molecule data would differ in vivo. However, the approaches used provide a way to observe more direct microbial activities and determine the impact of dietary ingredients on the microbial community and associated metabolites.

## 5. Conclusions

In conclusion, this small study exploring the impact of dietary intake of prebiotics and flavonoids observed improvements in cognitive performance, specifically in aspects of episodic memory and EF domains. The cognitive changes observed were concomitant with positive improvements in the gut microbiota. The in vitro work suggested that inulin and WBB may work in different ways, but both were able to lead to changes in the microbiota and metabolites. In the case of inulin, this included increases in the neuroactive molecule GABA. This pilot study, combined with the in vitro work, highlights the potential for the microbiota to be a key operator in cognitive outcomes; however, it is also clear that other pathways by which foods impact cognitive function are at play.

## Figures and Tables

**Table 1:** Characteristics of freeze-dried blueberry products.

Compound	Composition
Total polyphenols	2900 mg/100 g
Anthocyanins	1900 mg/100 g
Procyanidins	Not quantified
Vitamin C	335 mg/100 g
Total sugars	70 g/100 g
Fructose	36 g/100 g
Glucose	34 g/100 g
Dietary fibre	16 g/100 g
Insoluble	18 g/100 g [14]
Soluble	15.2 g/100 g [14]

**Table 2:** Oligonucleotide probes used in this study for FISH-FCM analysis of bacterial populations. +: These probes are used together in equimolar concentration of 50 ng/μL.

Probe Name	Sequence (3′–5′)	Target Group	Reference
Non Eub	ACTCCTACGGGAGGCAGC	Negative control	[30]
Eub338 I +	GCT GCC TCC CGT AGG AGT	Most bacteria	[31]
Eub338 II +	GCA GCC ACC CGT AGG TGT	Planctomycetales	[31]
Eub338 III +	GCT GCC ACC CGT AGG TGT	Verrucomicrobiales	[31]
Bif164	CAT CCG GCA TTA CCA CCC	Most *Bifidobacterium* spp. and *Parascardovia denticolens*	[32]
Lab158	GGTATTAGCAYCTGTTTCCA	Most *Lactobacillus*, *Leuconostoc*, and *Weissella* spp., *Lactococcus lactis*, all *Vagococcus*, *Enterococcus*, *Melisococcus*, *Tetragenococcus*, *Catellicoccus*, *Pediococcus*, and *Paralactobacillus* spp.	[33]
Bac303	CCA ATG TGG GGG ACC TT	Most Bacteroidaceae and Prevotellaceae, some Porphyromonadaceae	[34]
Erec482	GCT TCT TAG TCA RGT ACCG	Most of the *Clostridium coccoides-Eubacterium rectale* group (*Clostridium* clusters XIVa and XIVb)	[35]
Rrec584	TCA GAC TTG CCG YAC CGC	*Roseburia* subcluster	[36]
Prop853	ATT GCG TTA ACT CCG GCAC	Clostridial cluster IX	[36]
Chis150	TTATGCGGTATTAATCTYCCTTT	Most of the *Clostridium histolyticum* group (*Clostridium* clusters I and II)	[35]
Ato291	GGT CGG TCT CTC AAC CC	*Atopobium*, *Colinsella*, *Olsenella* and *Eggerthella* spp., *Cryptobacterium curtum*, *Mycoplasma equigenitalium*, and *Mycoplasma elephantis*	[37]
Fprau655	CGCCTACCTCTGCACTAC	*Faecalibacterium prausnitzii* and related sequences	[38]
DSV687	TAC GGA TTT CAC TCC T	Most *Desulfovibrionales* (excluding *Lawsonia*) and many *Desulfuromonales*	[39]

**Table 3:** Demographic data for inulin, WBB and placebo participants.

	Inulin (*n* = 5)			WBB (*n* = 4)			Placebo (*n* = 4)		
	Mean	SD	Range	Mean	SD	Range	Mean	SD	Range
Age	9.20	1.30	7–10	8.00	1.41	7–10	8.50	1.29	7–10
BAS 3 ^a^	50.40	2.41	48–53	49.75	1.71	48–52	49.25	0.50	49–50
Gender (M: F)	3:2	-	-	3:1	-	-	1:3	-	-

^a^ British Ability Scale 3; measured against normal data (Swinson, 2013) [43].

**Table 4:** Bacterial groups (total bacteria, *Bifidobacterium* spp., *Lactoabacillus-Enterococcus* spp., *Bacteroides–Prevotella* spp., *Clostridium* clusters XIVa and XIVb and *Roseburia* subcluster) detected by FISH-FCM (Log_10_ cells/mL) enumerated from each vessel (V1, V2, and V3) in colonic model before (SS1) and after (SS2) the daily administration of inulin (0.6 g/d) and of WBB (1 g/d). Values are mean (*n* = 3) ± SD. Significant difference in each vessel: * *p* < 0.05; a indicates a significant difference compared to the other substrate at the same time point.

	Vessel	Steady State	Total Bacteria			*Bifidobacterium* spp.			*Lactobacillus* spp. and *Enterococcus* spp.			*Bacteroides* spp. and *Prevotella* spp.			*Clostridium* Clusters XIVa and XIVb			*Roseburia* Subcluster		
Inulin	1	1	8.95	±0.18		8.53	±0.35		7.36	±0.16		7.59	±0.58		8.36	±0.61		6.64	±0.24	
	2	1	8.86	±0.31		8.42	±0.30		7.31	±0.09		7.72	±0.57		7.86	±0.56		6.62	±0.09	
	3	1	8.83	±0.14		8.08	±0.08		7.34	±0.31		7.72	±0.54		8.31	±0.56		7.05	±0.06	
	1	2	9.06	±0.23		8.90	±0.19	* a	7.94	±0.49	* a	7.48	±0.69		7.28	±0.65		5.97	±0.16	
	2	2	9.08	±0.08		8.88	±0.13		7.86	±0.40		7.70	±0.57		7.26	±0.54		5.96	±0.21	
	3	2	8.96	±0.04		8.62	±0.38	* a	7.80	±0.36		7.34	±0.56		7.17	±0.55		6.29	±0.16	
WBB	1	1	8.65	±0.37		8.35	±0.67		7.25	±0.07		7.44	±0.28		7.62	±0.74		6.52	±0.71	
	2	1	8.73	±0.43		8.34	±0.86		7.19	±0.03		7.57	±0.06		8.00	±0.65		5.90	±0.34	
	3	1	8.49	±0.35		8.14	±0.14		7.02	±0.09		7.25	±0.22		7.26	±0.39		5.88	±0.76	
	1	2	8.77	±0.44		8.48	±0.56	* a	7.75	±0.20	* a	7.39	±0.27		7.60	±0.37		6.34	±0.62	
	2	2	8.66	±0.45		8.41	±0.61		7.48	±0.17		7.58	±0.40		7.80	±0.61		5.89	±0.48	
	3	2	8.63	±0.26		8.26	±0.28	a	7.45	±0.15		7.38	±0.35		7.37	±0.17		5.84	±0.36	

**Table 5:** Bacterial groups (*Atopobium* cluster, *Clostridium* clster IX, *Faecalibacterium prausnitzii*, Desulfonovibrionales and Desulfuromonales and *Clostridium* clusters I and II) detected by FISH-FCM (Log_10_ cells/mL) enumerated from each vessel (V1, V2, and V3) in colonic model before (SS1) and after (SS2) the daily administration of inulin (0.6 g/d) and of WBB (1 g/d). Values are mean (*n* = 3) ± SD. Significant difference in each vessel: * *p* < 0.05; a indicates a significant difference compared to the other substrate at the same time point.

	Vessel	Steady State	*Atopobium* Cluster			*Clostridium* Cluster IX			*Faecalibacterium prausnitzii*			Desulfovibrionales and Desulfuromonales			*Clostridium* Clusters I and II		
Inulin	1	1	8.34	±0.78		6.70	±0.28		6.38	±0.57		6.53	±0.11		6.23	±0.60	
	2	1	8.25	±0.64		6.67	±0.22		6.43	±0.59		6.73	±0.07		6.05	±0.43	
	3	1	8.29	±0.60		6.66	±0.16		6.85	±0.59		6.78	±0.10		6.19	±0.62	
	1	2	8.16	±0.80		6.39	±0.45		6.19	±0.61		6.20	±0.16		5.49	±0.44	
	2	2	7.82	±0.76		5.99	±0.06		5.97	±0.21		6.18	±0.19		5.67	±0.57	
	3	2	8.02	±0.88		6.45	±0.47		6.18	±0.38		6.32	±0.34		6.03	±0.04	
WBB	1	1	7.93	±0.75		6.93	±0.14		6.75	±0.99		6.37	±0.23		6.40	±0.56	
	2	1	8.04	±0.57		6.80	±0.27		6.57	±0.83		6.47	±0.55		6.20	±0.40	
	3	1	7.66	±0.43		6.41	±0.18		6.16	±0.99		6.38	±0.19		6.01	±0.32	
	1	2	7.77	±0.43		6.61	±0.52		6.81	±0.69		6.53	±0.41		5.96	±0.80	
	2	2	7.99	±0.39		6.51	±0.31		6.70	±0.48		6.34	±0.20		5.84	±0.58	
	3	2	7.66	±0.14		6.57	±0.30		6.67	±0.56		6.26	±0.42		5.92	±0.52	

**Table 6:** Impact of interventions on cognitive outcome variables.

	Baseline			Post-Intervention			Post-Washout		
	Inulin (*n* = 5)	WBB (*n* = 4)	Placebo (*n* = 4)	Inulin (*n* = 5)	WBB (*n* = 4)	Placebo (*n* = 4)	Inulin (*n* = 5)	WBB (*n* = 4)	Placebo (*n* = 4)
**RAVLT**									
Word Span	5.2 (1.10)	4.5 (1.00)	3.75 (0.96)	6.4 (1.69)	5.25 (0.96)	4.75 (1.50)	7.2 (2.49)	5.25 (0.50)	4.75 (0.96)
Total Acquisition	42.6 (8.44)	36.25 (7.23)	35.5 (5.51)	48.8 (10.89) *	40.25 (7.23)	31.25 (5.56)	52.21 (8.15) *	38.5 (3.70)	29.25 (3.40)
Final Acquisition	9.4 (2.88)	9.25 (1.50)	6.75 (1.26)	10.22 (2.95) *	10.57 (1.41) *	5.75 (0.96)	12.16 (1.41) *	9.25 (0.50) *	6.25 (0.96)
Total Amount Learned	4.2 (1.92)	4.75 (2.06)	3.07 (1.83)	3.8 (2.17)	4.75 (0.96)	2 (0.82)	4.8 (2.17)	4 (0.01)	1.5 (1.00)
Delayed Recall	6.4 (2.61)	5.16 (3.27)	5.03 (0.82)	9.61 (1.82) *	9.53 (1.29) *	4.17(0.82)	9.26 (1.58)	7.33 (0.82)	5.48 (0.82)
Word Recognition									
Yes Trials	10 (2.92)	14.5 (2.08)	9.25 (3.30)	17.6 (6.66)	20 (6.06)	19.25 (4.99)	17.2 (5.76)	11 (4.08)	20.25 (6.18)
No Trials	39 (13.73)	36.5 (20.95)	29.5 (5.80)	86.4 (5.98)	98.25 (2.22)	94.25 (5.74)	77.2 (22.35)	86.5 (17.82)	80.75 (16.52)
**MANT**									
**Accuracy**									
Congruent High Load	0.55 (0.28)	0.44 (0.42)	0.25 (0.06)	0.73 (0.10)	0.81 (0.13)	0.59 (0.14)	0.67 (0.12)	0.81 (0.06) *	0.59 (0.11)
Congruent medium Load	0.59 (0.29)	0.45 (0.39)	0.22 (0.14)	0.68 (0.19) *	0.56 (0.12)	0.41 (0.11)	0.75 (0.07)	0.77 (0.09)	0.73 (0.04)
Incongruent High Load	0.48 (0.31)	0.41 (0.05)	0.34 (0.19)	0.32 (0.10)	0.47 (0.14)	0.43 (0.10)	0.36 (0.19)	0.40 (0.13)	0.51 (0.19)
Incongruent Medium Load	0.48 (0.29)	0.35 (0.18)	0.37 (0.10)	0.26 (0.22)	0.24 (0.16)	0.26 (0.11)	0.27 (0.18)	0.34 (0.11)	0.41 (0.13)
Neutral Low Load	0.58 (0.27)	0.24 (0.22)	0.19 (0.26)	0.31 (0.05)	0.41 (0.10)	0.66 (0.33)	0.44 (0.14)	0.40 (0.04)	0.31 (0.03)
**Reaction Time (RT)**									
Congruent High Load	701.65 (102.81)	577.09 (162.95)	708.42 (117.42)	743.09 (61.84)	644.17 (186.71)	755.97 (63.19)	768.22 (104.02)	686.97 (87.05)	791.82 (103.49)
Congruent medium Load	702.88 (105.53)	560.86 (171.07)	711.75 (119.68)	752.84 (68.62)	677.86 (126.32)	769.95 (65.78)	830.37 (91.58)	654.56 (116.85)	863.37 (62.67)
Incongruent High Load	674.19 (57.82)	666.57 (130.91)	687.55 (57.17)	772.12 (124.59)	708.57 (145.81)	805.72 (114.77)	759.51 (64.04)	709.80 (143.64)	782.98 (42.37)
Incongruent Medium Load	726.26 (85.54)	694.69 (172.72)	716.21 (95.31)	830 (117.55)	736.69 (153.39)	841.64 (132.37)	790.62 (110.35)	704.56 (128.19)	828.16 (82.72)
Neutral Low Load	686.77 (125.49)	584.74 (162.76)	693.47 (143.87)	767.56 (91.84)	676.74 (82.76)	790.21 (88.47)	782.06 (141.02)	665.18 (104.35)	843.75 (33.91)

Data are presented as mean (SD) data for inulin, WBB, and placebo participants’ performance on cognitive outcome variables (RAVLT, Serial 3s, word recognition, and MANT). Outcome measures are at baseline, post-intervention (4 weeks), and post-washout (8 weeks). Number of recall words from word span (A1), total acquisition (A1-A5), final acquisition (A5), delayed recall (A7), and total amount learned (A5-A1) from RAVLT among inulin, WBB, and placebo participants at baseline, post-intervention and post-washout periods. * Significantly different from the placebo group, *p* < 0.05 (LMM with baseline performance as covariate).

## Data Availability

All data generated are included within this manuscript.
